# Neural correlates of an early attentional capture by positive distractor words

**DOI:** 10.3389/fpsyg.2015.00024

**Published:** 2015-01-26

**Authors:** José A. Hinojosa, Francisco Mercado, Jacobo Albert, Paloma Barjola, Irene Peláez, Cristina Villalba-García, Luis Carretié

**Affiliations:** ^1^Instituto Pluridisciplinar, Universidad Complutense de MadridMadrid, Spain; ^2^Facultad de Psicología, Universidad Complutense de MadridMadrid, Spain; ^3^Facultad de Ciencias de la Salud, Universidad Rey Juan CarlosMadrid, Spain; ^4^Facultad de Psicología, Universidad Autónoma de MadridMadrid, Spain

**Keywords:** emotion, positive distractors, anterior N1, word processing, event-related potentials

## Abstract

Exogenous or automatic attention to emotional distractors has been observed for emotional scenes and faces. In the language domain, however, automatic attention capture by emotional words has been scarcely investigated. In the current event-related potentials study we explored distractor effects elicited by positive, negative and neutral words in a *concurrent but distinct target distractor paradigm*. Specifically, participants performed a digit categorization task in which task-irrelevant words were flanked by numbers. The results of both temporo-spatial principal component and source location analyses revealed the existence of early distractor effects that were specifically triggered by positive words. At the scalp level, task-irrelevant positive compared to neutral and negative words elicited larger amplitudes in an anterior negative component that peaked around 120 ms. Also, at the voxel level, positive distractor words increased activity in orbitofrontal regions compared to negative words. These results suggest that positive distractor words quickly and automatically capture attentional resources diverting them from the task where attention was voluntarily directed.

## INTRODUCTION

In order to maintain coherent behavior in a continuously changing environment, attentional processes are controlled endogenously to allow for keeping goal-directed behaviors in spite of distracting events. At the same time, organisms need to be able to effectively process novel, unexpected events, that could be either advantageous or dangerous, so as to ensure appropriate responses with either approach or avoidance behavior (Egeth and Yantis, 1997; Chica et al., 2013). The mechanism that is able to detect the appearance of these new events is called exogenous attention (also referred to as bottom-up, involuntary or stimulus-driven attention). It may be described as an adaptive mechanism for the rapid detection and processing of biologically relevant events, even when individuals are engaged in a resource-consuming task (Carretié, 2014). Exogenous shifts are reflexive, with attention being automatically pulled by external stimulation. According to different theoretical views (see Yantis, 2000 for a review), exogenous attention involves several processes such as the spatial automatic orientation of processing resources toward those events that deserve further processing (Sokolov, 1963; Graham and Hackley, 1991; Corbetta and Shulman, 2002; Posner et al., 2007), or the modulation of perceptual neural mechanisms that potentiate the processing of those stimuli capturing attention (Serences and Yantis, 2007; Asplund et al., 2010).

The results of several event-related potential (ERP) studies have revealed that some components may be related to distinct mechanisms involved in exogenous attention. In this sense, an anterior N1component peaking around 100 ms has been associated with an attentional mechanism of the prefrontal cortex, which directs attention and generates a bias signal that either enhances or suppresses sensory representations in visual pathways (Hillyard and Anllo-Vento, 1998; Barceló et al., 2000; Di Russo et al., 2003). Perceptual potentiation seems to be reflected by modulations in posterior P1and N1 components peaking around 100 and 150 ms, respectively, (Hillyard et al., 1998; Vogel and Luck, 2000; Di Russo et al., 2005; Natale et al., 2006). Several dorsal and ventral brain areas in the frontal and parietal cortex have been proposed to subserve attentional networks implicated in exogenous attention (see Corbetta and Shulman, 2002, and Corbetta et al., 2008 for reviews). These regions seem also to exert a modulatory control over the activity of occipital visual cortices (Kastner et al., 1998; Brefczynski and DeYoe, 1999).

Emotional stimuli are particularly relevant for an organism’s survival. Indeed, enhanced shifts in attention from the target stimulus toward competing emotional as compared to neutral faces or scenes presented as distractors are consistently observed (see Carretié, 2014, and Pourtois et al., 2013, for reviews). Capture of exogenous attention by emotional distractors increases reaction times and/or errors (e.g., Schimmack and Derryberry, 2005; Hodsoll et al., 2011). Also, depending on task demands and the current stimuli used, modulations by emotional compared to neutral distractors affect relatively early and/or late ERP components, including the P1 and the N1, as well as the so-called early posterior negativity (EPN), the P2 and the Late Positive Component late positive component (LPC; e.g., digit-categorization tasks: Carretié et al., 2009; perceptual discrimination tasks: Doallo et al., 2006; De Cesarei et al., 2009; Pourtois et al., 2010). Finally, studies providing spatial information on brain activity have revealed the involvement of visual cortices and fronto-parietal attentional networks in the processing of task-irrelevant emotional stimuli (e.g., Vuilleumier et al., 2001; Pessoa and Ungerleider, 2004; Mitchell et al., 2007; Carretié et al., 2012).

Correlates of exogenous attentional capture by emotional task-irrelevant stimuli have been also observed in the language domain, although the mechanisms that operate for the processing of verbal distractors have been much less explored than for pictorial materials. Several studies have used experimental paradigms in which targets and emotional distractor words were not concurrent in time, such as the dot probe task (e.g., MacLeod et al., 1986), affective variants of the cue-target paradigm (e.g., Stormark et al., 1995; Amir et al., 2003), or the attentional blink paradigm (e.g., Keil and Ihssen, 2004; Arnell et al., 2007). Overall, these studies have provided important information on exogenous attention processes, which suggests that emotional verbal distractors elicited an involuntary capture of attention. However, some limitations have been noted since orienting toward and disengaging from a stimulus are processes that may be difficult to differentiate in these paradigms (Salemink et al., 2007; Cisler et al., 2009). Similar concerns have been raised about using tasks in which emotional distractors and targets engaging voluntary attention are not physically segregated. Examples of these paradigms are those exploring the emotional Stroop effect (e.g., McKenna and Sharma, 1995; Thomas et al., 2007; González-Villar et al., 2014), those using affective lexical decision tasks (Hofmann et al., 2009; Hinojosa et al., 2010b), or those where specific non-emotional aspects (e.g., letter font detection) of words have to be identified (e.g., Schacht and Sommer, 2009a; Hinojosa et al., 2014). It has been claimed that these tasks may not trigger some of the processes involved in exogenous attention, such as spatial reorienting mechanisms (Carretié, 2014). Indeed, they have been most commonly used to explore lexical or conflict-related processes rather than exogenous attention.

A different source of evidence comes from studies using *concurrent but distinct target distractor paradigms* (CDTD) or *directed attention tasks* (MacNamara et al., 2013; Carretié, 2014). In these tasks, elements on the screen to which voluntary attention must be directed to perform a task (targets) and elements that are task-irrelevant (distractors) appear at the same time but are physically segregated. The use of CDTD tasks may be a suitable tool to explore exogenous attention mechanisms since both orienting of attention and sensory enhancement processes seem to be operating in these paradigms (Carretié, 2014). To the best of our knowledge, however, only three studies have compared the processes triggered by emotional and neutral distractor words with CDTD tasks (see also Rampone et al., 2014, who did not include neutral distractor words). The results of these studies suggest that emotional words capture attention to a lesser extent than do scenes or faces (Carretié, 2014), which are in line with reports showing differences in the processing of emotional pictorial and verbal stimuli (Hinojosa et al., 2009; Schacht and Sommer, 2009b; Frühholz et al., 2011; Schlochtermeier et al., 2013). In this sense, Harris and Pashler (2004) found slowed reaction times to negative distractors only after the first presentation of task-irrelevant words using a digit categorization task. Also with this paradigm, Aquino and Arnell (2007) reported increased reaction times to sexually explicit distractors compared to neutral words, but not between threatening or school-related items and neutral words. Finally, Trauer et al. (2012) used a visual foreground perceptual task to investigate distraction effects by emotional words on steady-state visual evoked potentials (SSVEPs). Behavioral data and SSVEP amplitudes showed no differences regardless of the emotional content of distractor words, which was taken to suggest an absence of attentional modulation in early visual areas. Lexico-semantic effects in middle and late latency ERP components were also explored. The authors found enhanced amplitudes in the P2 and N400 components to negative task-irrelevant words and concluded that emotional distractor words captured lexico-semantic processing resources.

The heterogeneity of the findings suggests that more studies are needed in order to clarify how the distinct processes involved in exogenous attention modulate the processing of task-irrelevant emotional words. In this sense, compared with behavioral measures, ERPs make it possible to determine which stages are being affected by a specific experimental manipulation. Another advantage over behavioral methods is that they can provide a measure of processing stimuli even when there is no behavioral change. In the only prior ERP study, Trauer et al. (2012) focused their ERP analyses on the stage of elaborated meaning evaluation -P2, N400, and LPC components- due to some limitations of the SSVEP procedures to explore early latency components. Thus, the involvement of orienting mechanisms and/or enhanced sensory processing that occur at early attentional processing stages remains still unexplored with ERPs. The present study sought to clarify the mechanisms involved in exogenous attention to verbal stimuli. To this end, emotional and neutral words were presented as distractors while participants carried out a demanding digit categorization CDTD task. We expected effects to arise in those ERP components that have been associated with the automatic orientation of processing resources and/or the modulation of perceptual neural mechanisms in prior literature, namely the P1 and the N1 (Hillyard et al., 1998; Di Russo et al., 2005). Additionally, we examined those components - the P2, the EPN, the N400, and the LPC- that have been modulated by emotional content in word processing studies with a variety of experimental paradigms including lexical decision tasks (Kanske and Kotz, 2007; Scott et al., 2009; Méndez-Bértolo et al., 2011), silent reading (Kissler et al., 2007, 2009; Herbert et al., 2008), structural decision tasks (i.e., identification of italicized letters, Schacht and Sommer, 2009a), or grammatical decision tasks (i.e., counting of nouns or adjectives, Kissler et al., 2009).

As a second goal, we explored the neural origin of exogenous attention to emotional distractor words, a question that has not been addressed in previous research. To this aim, source location analyses were performed using exact low resolution brain electromagnetic tomography (eLORETA; Pascual-Marqui, 2007). According to previous literature, activation of those brain regions underlying attentional networks and emotional processing was hypothesized, namely frontal, parietal and/or extrastriate visual cortices (Vuilleumier, 2005).

## MATERIALS AND METHODS

### PARTICIPANTS

Thirty undergraduate students (23 females and 7 males) from the *Universidad Rey Juan Carlos*, with an age range between 18 and 26 (mean = 18.96, SD = 1.92), participated in this experiment. Participants were native speaker of Spanish and right-handed, as assessed with the Edinburgh Handedness Inventory (Oldfield, 1971): LQ > +72. All subjects gave written informed consent and reported normal or corrected-to-normal visual acuity. The study was approved by the Ethics Committee of the *Universidad Rey Juan Carlos*.

### STIMULI AND PROCEDURE

Three types of distractor words were presented to participants in a digit categorization task: negative, positive and neutral words. The complete set of verbal stimuli consisted of 150 Spanish nouns (50 per emotional category). These words were selected from a pilot study that comprised 720 nouns. In this study, 45 individuals (different from those participating in the current study) rated valence, arousal, and the level of concreteness of each word on a 9-point Likert scale (for a detailed description of the pilot study see Hinojosa et al., 2009). Equal numbers of negative, positive and neutral distractor words were selected according to several criteria that were contrasted with analyses of variance (ANOVAs; see **Table 1**): (a) negative and positive words were matched in arousal rating but both differed from neutral words; (b) negative, positive and neutral nouns differed in valence ratings; (c) all nouns had similar concreteness, word length and frequency of use (Alameda and Cuetos, 1995). **Table 1** summarizes mean values in arousal, valence and concreteness for nouns, as well as mean word frequency and word length.

**Table 1:** Means and SD of valence (1 highly unpleasant, 9 highly pleasant), arousal (1 highly calming, 9 highly arousing), concreteness (1 highly abstract, 9 highly concrete), frequency of use (per one million), number of syllables, and number of letters.

	Valence	Arousal	Concreteness	Frequency	Syllables	Letters
Negative	2.13 (0.5)	7.29 (0.5)	6.10 (1.3)	80.72 (108)	2.88 (0.8)	7.08 (2)
Neutral	5.06 (0.1)	5.07 (0.2)	6.11 (1.8)	84.36 (108)	2.96 (0.8)	6.84 (2)
Positive	7.67 (0.5)	7.23 (0.5)	6.07 (1.4)	85.14 (101.1)	2.96 (0.8)	7.02 (2)
**ANOVA**
Emotion		*F* = 2117.7*	*F* = 421.7*	*F* = 0.0^ns^	*F* = 0.0^ns^	*F* = 0.1^ns^	*F* = 0.1^ns^	

d.f. = 2,98; *n.s.*, non-significant, *p < 0.001.

**FIGURE 1:**
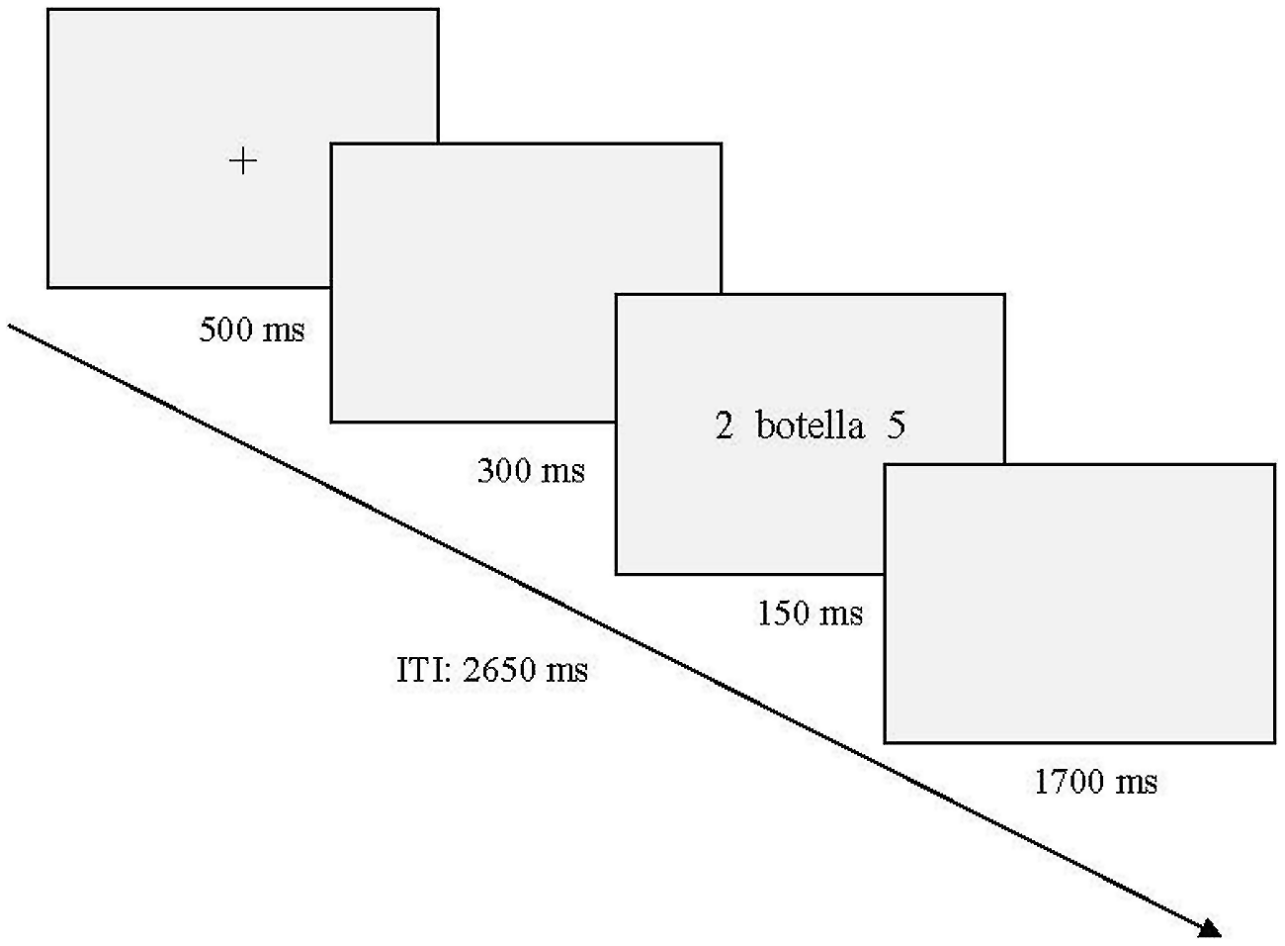
**Schematic representation of the sequence described in the main text.** An example belonging to neutral words during discordant digit condition is represented (“botella” = *bottle*). ITI, Intertrial interval.

Participants sat in an electrically and acoustically isolated room in a comfortable chair. The stimuli were presented on a computer monitor that was positioned at eye level about 60 cm in front of the participant. Words were presented in lower case letters at fixation with digits in the left and the right periphery (10° eccentricity). The size of all words ranged between 7.64 and 2.86° (width) × 0.95° (height). Only digits from 2 to 8 were used (0.95° height). Words and digits appeared in black against a light gray background. The sequence of events in each trial is represented in **Figure 1**. First, a fixation cross appeared in the center of the screen and remained there for 500 ms. This fixation cross was followed by a blank screen interval of 300 ms and then words flanked by the two digits were presented for 150 ms and were followed by a 1700 ms blank interval. The intertrial interval was 2650 ms.

As indicated, participants performed a digit categorization task. They were told to press, ‘as accurately and rapidly as possible,’ one key of a response device if both digits were either even or odd (i.e., if they were ‘concordant’), and a different key if one digit was even and the other was odd (i.e., if they were ‘discordant’). In half of the trials digits were concordant whereas they were discordant in the other half. The same combination of digits was repeated across emotional conditions in order to ensure that task demands were identical in trials with negative, positive and neutral distractors. The order of presentation of the 150 trials (50 trials for each of the three emotional categories) was pseudorandomized so no more than three consecutive trials of the same emotional or numerical category appeared consecutively. Stimuli were presented in two runs of 75 stimuli with a brief resting period between them. Participants were requested to avoid blinking as much as they could. A training block of nine trials was provided at the beginning of the session to familiarize participants with the task.

### EEG RECORDING AND PRE-PROCESSING

Continuous electroencephalographic (EEG) activity was recorded using an electrode cap (ElectroCap International) with 60 homogeneously distributed scalp electrodes. All electrodes were referenced to the linked mastoids. Electrooculographic (EOG) data were recorded supra- and infraorbitally (vertical EOG), as well as from the left versus right orbital rim (horizontal EOG). Electrode impedances were kept below 5 kΩ. An online bandpass filter from 0.1 to 40 KHz was used (3 dB points for -6 dB/octave roll-off), and digitization sampling rate was set to 250 Hz. Off-line pre-processing was performed using Brain Vision Analyzer software (Brain Products). The continuous EEG recording was divided into 1000-ms epochs for each trial, beginning 200 ms before stimulus onset. Baseline correction was made using the 200-ms period prior to the onset of stimulus. Trials in which subjects responded erroneously or did not respond were eliminated. EOG-artifact removal was carried out following the procedure described by Gratton et al. (1983). A careful EEG visual inspection was then performed in which epochs with artifacts were eliminated from further analyses. This artifact and error rejection procedure led to an average admission of 86.6% positive, 90% neutral, and 91.8% negative trials. The ERP averages were categorized according to each distractor category (negative, neutral, and positive).

### DATA ANALYSIS

#### Behavioral analysis

Mean reaction times (RTs) of correct responses and error rates (omissions and commissions) were analyzed. Repeated-measures ANOVAs on each measure were carried out with respect to Distractor type (three levels: negative, neutral, and positive). The Greenhouse–Geisser epsilon correction was applied when the assumption of sphericity was violated. *Post hoc* pairwise comparisons were two-tailed, paired-samples *t-tests* with Bonferroni correction for multiple comparisons. As a measure of effect size, partial η -square ($\eta_{p}^{2}$) is reported for significant effects.

#### ERP analysis

Detection and quantification of ERP components was carried out through covariance-matrix-based temporal principal component analysis (tPCA). All analyses were performed using IBM SPSS v20. The main advantage of tPCA over traditional procedures based on visual inspection of recordings and on ‘temporal windows of interest’ is that it presents each ERP component separately and with its ‘clean’ shape, extracting and quantifying it free of the influences of adjacent or subjacent components (Chapman and McCrary, 1995; Dien and Frishkoff, 2005). Indeed, the waveform recorded at a site on the head over a period of several 100 ms represents a complex superposition of different overlapping electrical potentials. Such recordings can stymie visual inspection. In brief, tPCA computes the covariance between all ERP time points, which tends to be high between those time points involved in the same component, and low between those belonging to different components. The solution is therefore a set of independent factors made up of highly covarying time points, which ideally correspond to ERP components. *Temporal factor scores*, the tPCA-derived parameter in which extracted temporal factors (TFs) may be quantified, is linearly related to amplitude. In the present study, the decision on the number of components to select was based on the scree test (Cattell, 1966). Extracted components were submitted to Promax rotation, as recommended (Dien, 2010, 2012).

Given that signal overlapping may occur also at the space domain, we performed subsequent spatial PCAs on every temporal factor. At any given time point, several neural processes (and hence, several electrical signals) may concur, and the recording at any scalp location at that moment is the electrical balance of these different neural processes. While temporal PCA “separates” ERP components along time, spatial PCA (sPCA) separates ERP components along space, each spatial factor ideally reflecting one of the concurrent neural processes underlying each temporal factor. Additionally, sPCA provides a reliable division of scalp into different recording regions, an advisable strategy prior to statistical contrasts, since ERP components frequently behave differently in some scalp areas than in others (e.g., they present opposite polarity or react differently to experimental manipulations). This method of analysis is reference-independent since the configuration of the scalp topography is independent of the reference electrode position (Pourtois et al., 2008). Basically, each region or spatial factor is formed with the scalp points where recordings tend to covary. As a result, the shape of the sPCA-configured regions is functionally based, and scarcely resembles the shape of the geometrically configured regions defined by traditional procedures. Moreover, each spatial factor can be quantified through the *spatial factor score*, a single parameter that reflects the amplitude of the whole spatial factor. Also in this case, the decision on the number of factors to select was based on the scree test, and extracted factors were submitted to Promax rotation.

Finally, repeated-measures ANOVAs on temporospatial factor scores were carried out with respect to Distractor type (three levels: negative, neutral, and positive). The Greenhouse–Geisser epsilon correction was applied when the assumption of sphericity was violated, and *post hoc* pairwise comparisons were two-tailed, paired-samples *t*-tests with Bonferroni correction for multiple comparisons. Effect sizes were also reported using the partial η -square ($\eta_{p}^{2}$) method.

#### Source localization analysis

In order to three-dimensionally locate the cortical regions that were sensitive to the experimental effects observed at the scalp level, exact low-resolution brain electromagnetic tomography (eLORETA; Pascual-Marqui, 2007; Pascual-Marqui et al., 2011) was applied to relevant temporal factor scores. eLORETA is a 3D, discrete linear solution for the EEG inverse problem, which provides inverse solutions that are reference-independent (Pascual-Marqui et al., 2011; Michel and Murray, 2012). Although, in general, solutions provided by EEG-based source-location algorithms should be interpreted with caution due to their potential error margins, LORETA solutions have shown significant correspondence with those provided by hemodynamic procedures in the same tasks (Dierks et al., 2000; Vitacco et al., 2002; Mulert et al., 2004). Moreover, the use of tPCA-derived factor scores instead of direct voltages (which leads to more accurate source-localization analyses: Dien et al., 2003, 2004; Carretié et al., 2004), contribute to reducing this error margin. In its current version, eLORETA computes the current density at each of 6239 voxels mainly located in the cortical gray matter of the digitized Montreal Neurological Institute (MNI) standard brain.

Specifically, three-dimensional current–density estimates for relevant temporal factor scores were computed for each participant and each experimental condition. Subsequently, the voxel-based whole-brain eLORETA-images (6239 voxels) were compared between conditions using the non-parametric mapping (SnPM) tool, as implemented in the sLORETA/eLORETA software package. As explained by Nichols and Holmes (2002), the non-parametric methodology inherently avoids multiple comparison-derived problems and does not require any assumption of normality. Voxels that showed significant differences between conditions (log-F-ratio statistic, two-tailed corrected *p* < 0.05) were located in anatomical regions and Brodmann areas (BAs).

## RESULTS

### BEHAVIORAL RESULTS

Average values for RTs, omission and commission error rates to each emotional word category are shown in the **Table 2**. Three repeated-measures ANOVAs were conducted on RTs, omission and commission error rates including Distractor type as a factor Although RTs for positive distractor trials were slower than for the rest of trials, statistical analyses did not reach significance [*F*(2,58) = 0.883, *p* = 0.372]. Also, no significant results were found for error rates [*F*(2,58) = 1.715, *p* = 0.191, for omissions, and *F*(2,58) = 1.359, *p* = 0.265 for commissions].

**Table 2:** Means and SD (in parenthesis) of reaction times (RTs) and errors rates (commission/omission) to each word category (positive, negative, and neutral).

	Positive words	Negative words	Neutral words
RTs (ms)	822.72 (30.11)	795.79 (40.72)	799.40 (42.72)
Error rates (commission)	0.051 (0.031)	0.038 (0.030)	0.050 (0.041)
Error rates (ommission)	0.071 (0.095)	0.058 (0.084)	0.070 (0.085)

### ERP RESULTS

**FIGURE 2:**
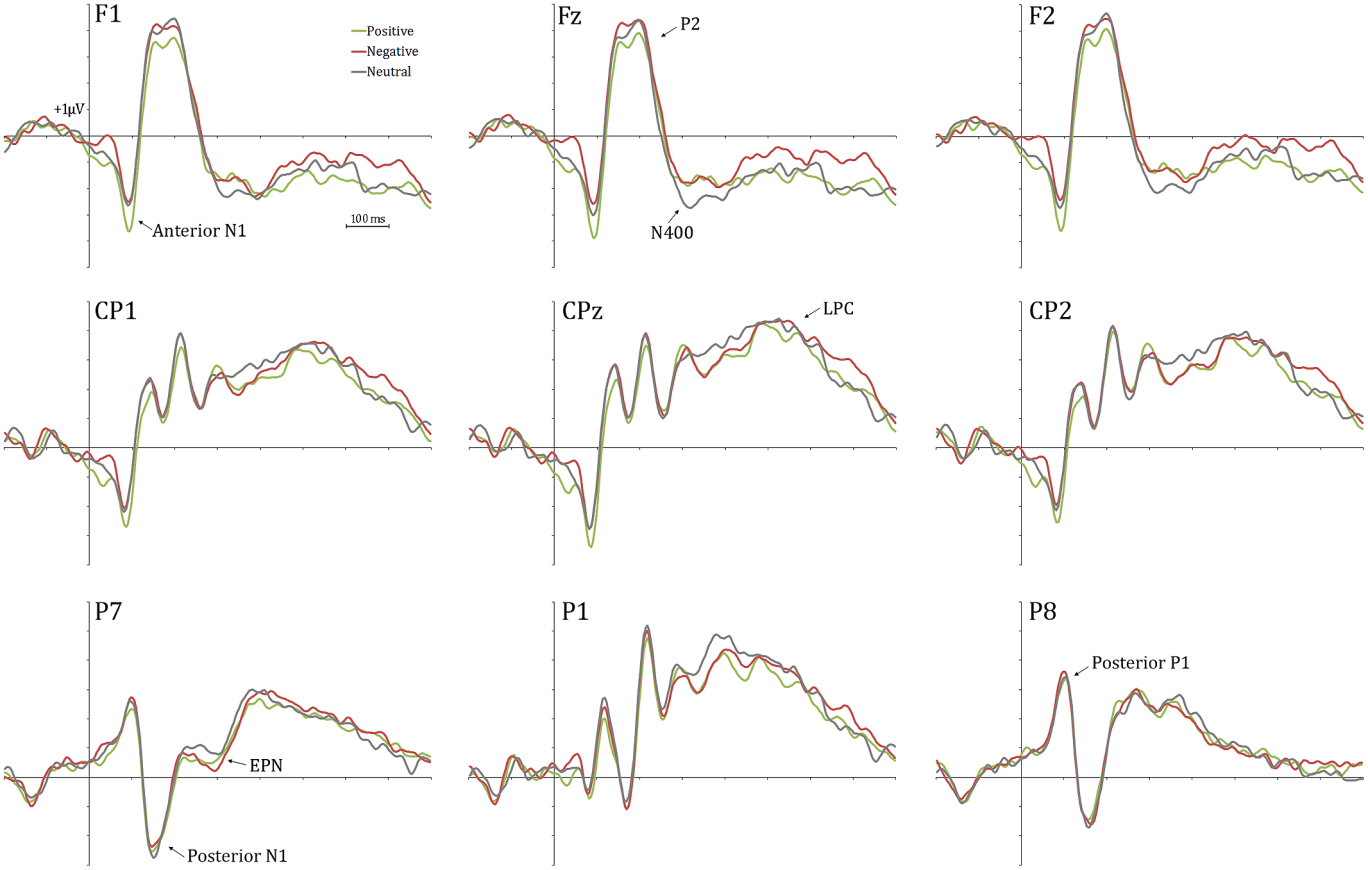
**Grand averages at a selection of electrodes.** Experimental effects are clearly visible at frontal electrode sites (anterior N1).

**FIGURE 3:**
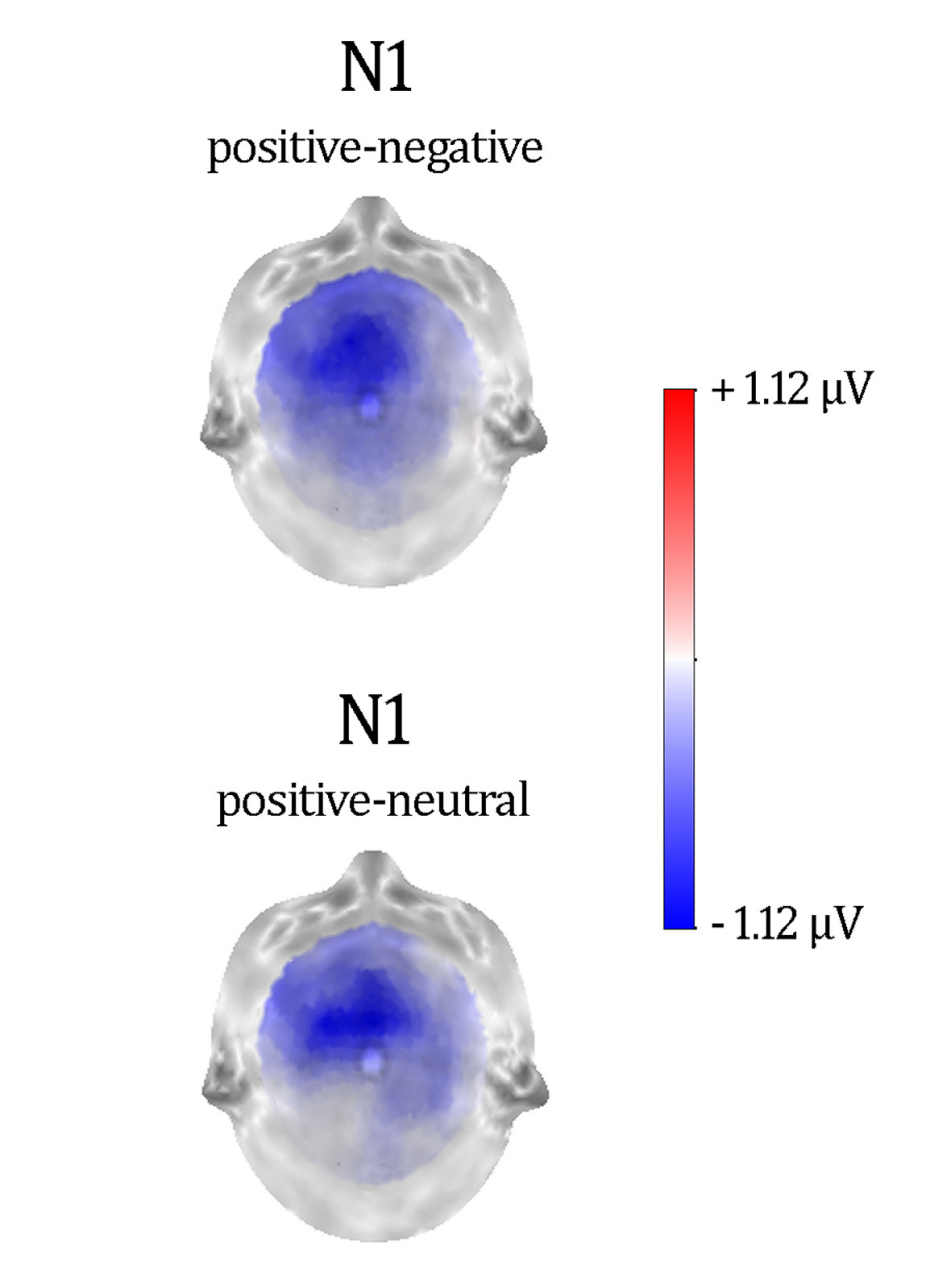
**Difference maps showing distractor type effects in N1.** These maps were computed at the peak latency of N1.

**Figure 2** shows a selection of grand averages once the baseline value (prestimulus recording) was subtracted from each ERP. As described later, experimental effects were observed at around 120 ms (N1) over anterior electrode positions (see F1 and F2 locations). **Figure 3** represents the topographic distribution of this effect.

**FIGURE 4:**
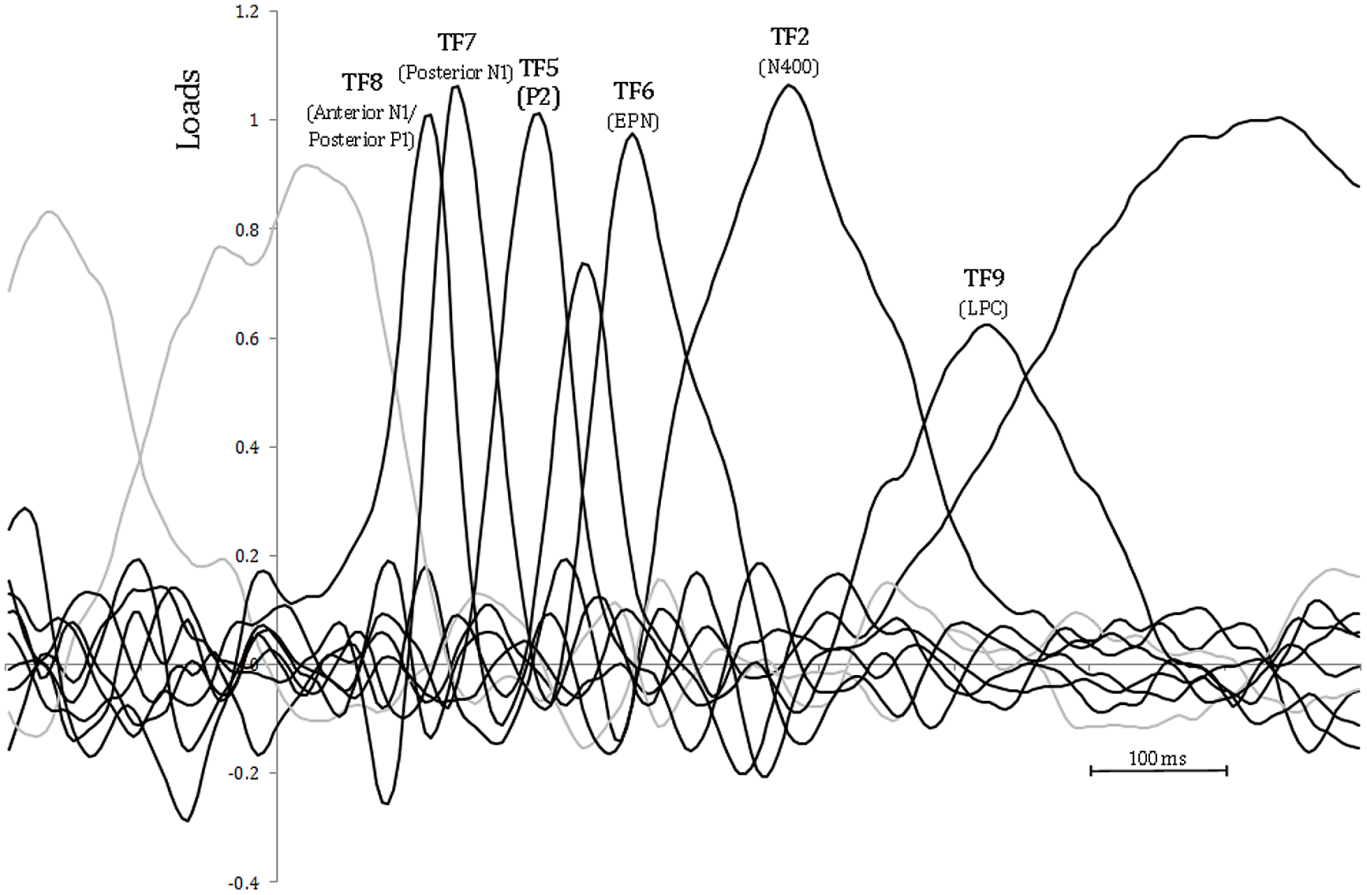
**Temporal principal component analysis (tPCA): factor loadings after Promax rotation.** Peak-latency of relevant factors is shown.

As a consequence of the application of the tPCA, several TFs were extracted from the ERPs (see **Figure 4**). Factor peak-latency and topography characteristics revealed TF8 as the component being associated with both posterior P1 and anterior N1, which typically overlap in time (Di Russo et al., 2003). Indeed, tPCA revealed that the two components were evoked at the same latency (peaking at 120 ms). However, differential characteristics of the posterior P1and the anterior N1 were patent both at the polarity and the scalp topography (as described later, see also **Figure 3**). Furthermore, TF7 (peaking at 140 ms), TF5 (peaking at 192 ms), TF6 (peaking at 270 ms), and TF2 (peaking at 380 ms) were related to posterior N1, P2, EPN, and N400 components, respectively. Finally, the LPC was decomposed in two centroparietal factors: TF9, peaking at 525 ms, and TF1, peaking at 730 ms.

As can be observed in **Table 3**, the sPCA decomposed TF8 in one anteriorly distributed factor (corresponding to the anterior N1) and two factors with posterior distributions (corresponding to the P1). Also, sPCA extracted three spatial factors for each of the remaining TFs. Therefore, the temporospatial PCA yielded a total of 24 factor combinations (three spatial factors extracted for each of 8 TFs).

**Table 3:** Description and statistical results for the factors extracted by temporospatial principal component analysis.

Temporal factor	Peak (ms)	Spatial factor	Scalp distribution	ANOVAs (Distractor type, d.f. = 2, 58)
TF8 (anterior N1/posterior P1)	110	SF1	Frontocentral	*F* = 5.04, *p* = 0.01,$\eta_{p}^{2}$ = 0.15
		SF2	Centroparietal	*F* = 0.35, *p* = 0.7
		SF3	Occipitoparietal	*F* = 0.15, *p* = 0.8
				
FT7 (posterior N1)	140	SF1	Frontocentral	*F* = 1.12, *p* = 0.3
		SF2	Parietooccipital (right)	*F* = 0.92, *p* = 0.4
		SF3	Parietooccipital (left)	*F* = 0.52, *p* = 0.6
				
FT5 (P2)	190	SF1	Frontal	*F* = 1.04, *p* = 0.4
		SF2	Parietooccipital	*F* = 0.1, *p* = 0.9
		SF3	Centroparietal	*F* = 1.65, *p* = 0.2
				
FT6 (EPN)	270	SF1	Frontocentral	*F* = 0.98, *p* = 0.4
		SF2	Parietooccipital	*F* = 0.85, *p* = 0.4
		SF3	Temporoparietal	*F* = 1.6, *p* = 0.2
				
FT2 (N400)	380	SF1	Frontal	*F* = 1.59, *p* = 0.2
		SF2	Occpitoparietal	*F* = 1.55, *p* = 0.2
		SF3	Centroparietal	*F* = 0.78, *p* = 0.4
				
FT9 (LPC)	520	SF1	Frontal	*F* = 2.26, *p* = 0.1
		SF2	Centroparietal	*F* = 0.93, *p* = 0.4
		SF3	Occipitotemporal	*F* = 0.6, *p* = 0.5

*TF, temporal factor; SF, spatial factor; d.f., degrees of freedom.*

Repeated-measures ANOVAs on these temporospatial factors with respect to Distractor type (three levels: negative, neutral, and positive) were carried out as previously described. **Table 3** provides the statistical details of these analyses. As can be appreciated, the effect of Distractor type was only significant for the anterior N1. *Post hoc* tests with Bonferroni correction for multiple comparisons showed enhanced anterior N1 amplitudes for positive compared to neutral and negative distractor words (*p*s < 0.05). The anterior N1 amplitude did not differ between neutral and negative distractor words (*p* = 1). As **Table 3** shows, no significant effects were found on other ERP components.

### SOURCE LOCALIZATION RESULTS

**FIGURE 5:**
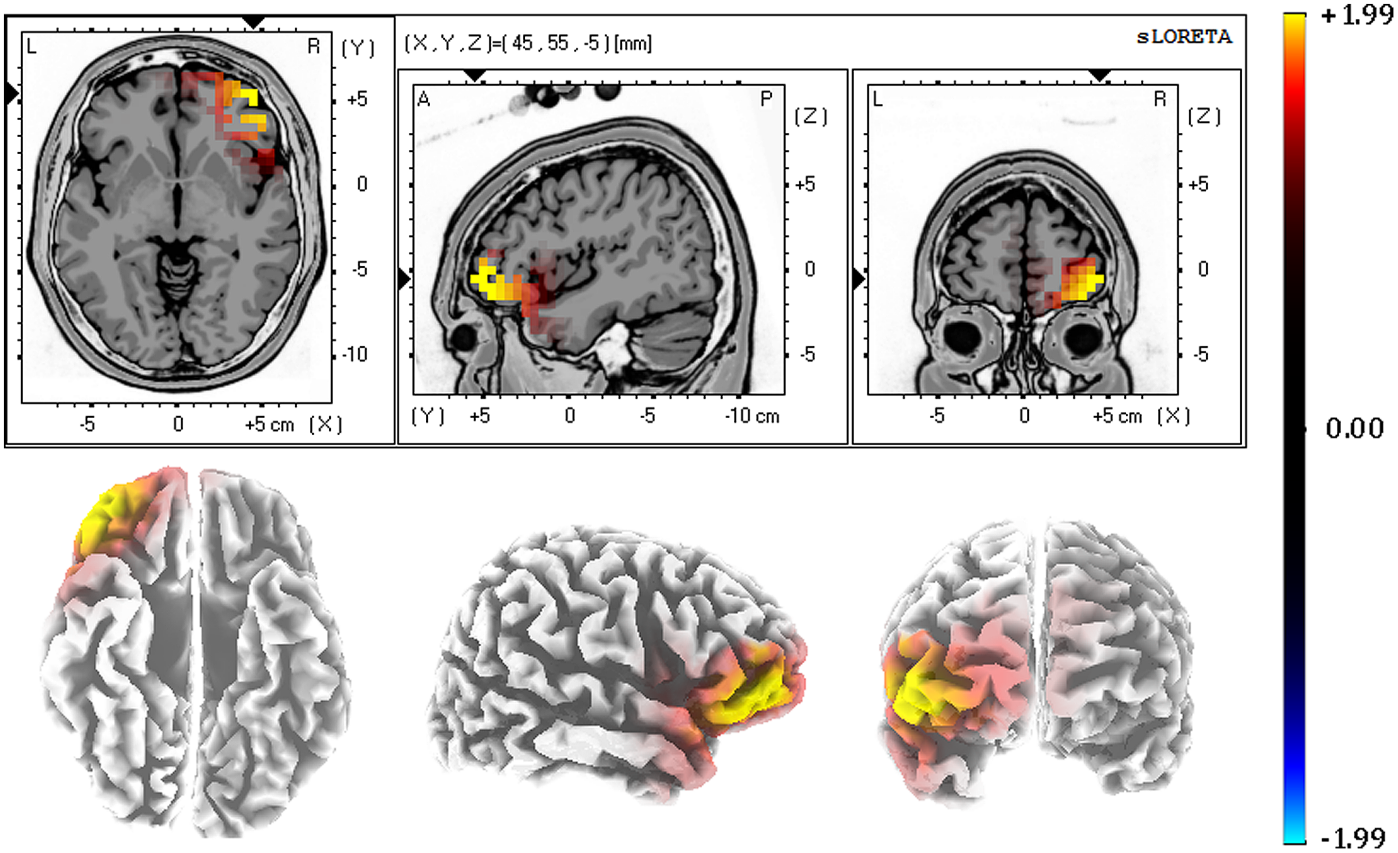
**Source localization results (eLORETA): increased N1-related activation to positive in comparison to neutral distractor words was observed in the orbitofrontal cortex.** Color bar represents voxel log-F ratio values. The threshold for corrected *p* < 0.05 was therefore 1.99. The strong yellow color indicates those voxels showing significant differences between conditions.

The last analytic step consisted of three-dimensionally localizing the cortical regions that were responsible for the differences observed in the anterior N1. To achieve this, N1 temporal factor scores of each subject, electrode, and condition were submitted to eLORETA. Then, the voxel-based whole brain eLORETA-images (6239 voxels) were compared between conditions using the SnPM approach. N1-related activation in response to positive distractor words was associated with enhanced activity compared to negative distractor words in several voxels. As illustrated in **Figure 5**, these voxels were located in the orbitofrontal cortex (OFC; peak MNI coordinates: *X* = 45, *Y* = 55, *Z* = -5; BAs 11/10/47). Activation differences between positive and neutral distractor words did not reach significance. Consistent with results from scalp ERPs, no activation differences were found between neutral and negative distractor words in any voxel.

## DISCUSSION

In the current study we investigated the processing of emotional distractor words while participants performed a digit categorization task. In line with previous studies using CDTD tasks, we did not observe any sign of attentional capture by emotional distractor words in behavioral measures. In this direction, Trauer et al. (2012) failed to report behavioral indices that evidenced the interference of emotional word content with a perceptual foreground task. Weak effects were found in other studies. In particular, delayed reaction times for emotional with respect to neutral distractor words have been reported only after the first occurrence of a negative word (Harris and Pashler, 2004), or for sexually explicit words (Aquino and Arnell, 2007). Since behavioral correlates of attentional capture by task-irrelevant emotional pictures and faces are usually observed (e.g., Vuilleumier et al., 2001; MacNamara and Hajcak, 2009; Calvo and Nummenmaa, 2011; Carretié et al., 2013b; but see Holmes et al., 2006; Carretié et al., 2013a), our data fits well with the idea that word distractors may be able to interrupt ongoing processing to a lesser extent than pictorial distractors (Carretié, 2014). Nonetheless, it should be remarked that behavioral measures are the final single output of a large set of neural processes that may not be always convergent. Notably, one advantage of using ERPs is that the components can be examined in the absence of an overt behavioral response (Luck, 2005). Indeed, current results corroborate the greatest sensitivity of ERPs to the effects of certain experimental manipulations. In this respect, neural results clearly showed that the emotional content of the distractor words modulated processing-resources devoted to a primary ongoing task, as suggested both by scalp and source-location data. In particular, positive distractor nouns compared to both neutral and negative distractor words were associated with enhanced amplitudes in an anteriorly distributed negative component peaking around 120 ms. Activity in the OFC was identified as the neural origin of this scalp-recorded component. Latency, amplitude and source-location analyses suggest that this component would be associated with attentional capture by positive distractor words. These results will be discussed in detail bellow.

As indicated in the Results section, a wave peaking around 120 ms after trial presentation was subdivided into two components by spatial principal component analyses. A posterior P1 deflection showed no amplitude differences between neutral and emotional distractors. Interestingly, however, positive distractor words elicited larger anterior N1 amplitudes than both negative and neutral task-irrelevant words. Similar modulations in a frontal N1 component for emotional task-irrelevant pictures have been recently found when participants’ attention was engaged in a counting task (Zhang et al., 2014). Prior studies linked this component to involuntary orientation of attention to relevant stimuli (Luck et al., 1993; Di Russo et al., 2003). Specifically, it has been suggested that the anterior N1 may reflect a prefrontal attentional mechanism that regulates sensory processing in visual cortices (Barceló et al., 2000; Pérez-Edgar and Fox, 2003).

The neural origin of our anterior N1, which seems to be generated in the OFC (BAs 11/10/47), argues in favor of the involvement of this region in attentional capture by positive distractor words. The OFC has been critically implicated in both the modulation of emotion and attentional control (Vuilleumier, 2005; Domínguez-Borrás and Vuilleumier, 2013). Neuroanatomical studies indicate that the OFC is reciprocally connected with the amygdala and extensive areas of prefrontal, motor and sensory cortices (Pandya and Yeterian, 1996; Cavada et al., 2000; Rolls, 2000). Specifically, it has been suggested that early activation of the OFC would modulate sensory cortices via direct feedback or indirect projections to attention and object-recognition systems in prefrontal, parietal and temporal cortices (Amaral et al., 2003; Vuilleumier, 2005). In particular, Bar et al. (2006) reported that object recognition elicited activity in the OFC around 130 ms and 50 ms before it developed in recognition-related fusiform regions. Also, activations to emotional cues in this prefrontal region have been reported around 120 ms, using intracranial (Kawasaki et al., 2001) and scalp recordings (Pourtois et al., 2004). In line with these findings, a recent proposal postulates that the medial part of the OFC is involved in the generation of affective predictions that initiate appropriate reactions to visual information, whereas the lateral regions of the OFC seems to be implicated in computing and sending predictions about the identity of visual stimuli to the visual system (Chaumon et al., 2014). Interestingly, enhanced activity in the OFC while exogenous attention is directed to task-irrelevant emotional pictures and faces has been previously reported (Vuilleumier et al., 2001; Bishop et al., 2004; Zhang et al., 2014). Thus, our current finding provides additional evidence supporting the implication of the OFC in exogenous attention to emotional verbal distractors, which may be triggered in part by the activation of predictive mechanism involving the processing of affective and identity-related information.

The selective enhancement of detection sensitivity to positive distractor words deserves further consideration. This finding agrees with the results of a growing body of research indicating that the OFC is a key structure in the neural circuitry of positive emotions and the processing of reward (Rolls, 2000; Burgdorf and Panksepp, 2006). In this direction, activation of the OFC has been found when mothers viewed pictures of their own compared to unfamiliar children (Nitschke et al., 2004), when participants received financial reward in a gambling task (Elliott et al., 2000), or when pleasant taste stimuli were delivered to participants (O’Doherty et al., 2001). Also, patients with OFC lesions responded faster to targets subsequent to positive distractors in a lateralized visual discrimination task (Hartikainen et al., 2012). Crucially, the results of an fMRI study by Lewis et al. (2007) showed a selective role of the OFC in the processing of valence during word processing. Thus, our data suggest activation in OFC seems to underlay selective attention to positive word distractors in CDTD tasks. Furthermore, the present results can be interpreted in terms of the positivity offset. This represents a tendency from the positive motivational system to respond more than the negative emotional system to comparably low levels of evaluative input, which seems to be the case of the processing of word distractors (Cacioppo et al., 1997; Ito and Cacioppo, 2005). Indeed, there is recent evidence indicating that as early as in the 80–120 ms time interval, the processing of positive and negative words implicates neural activity in different networks. Specifically, the processing of positive words was associated with activations in language and attention-related regions in left temporal, frontal and visual association cortices, whereas negative words activated the anterior cingulate cortex (Keuper et al., 2013). These effects were interpreted in terms of an “emotional tagging” of word forms associated to different processing strategies developed during language acquisition. These strategies include enhanced lexical processing of positive words and a fast language-independent alert response to negative words (Keuper et al., 2013). In agreement with this view, several studies reported valence-dependent effects at different processing stages that show facilitated lexical processing for positive words with both behavioral and ERPs measures (Kissler and Koessler, 2011; Kuchinke and Lux, 2012; Kissler and Herbert, 2013). This processing advantage has been linked to the orbitofrontal reward system (Kuchinke and Lux, 2012).

Resembling current findings, increased attentional capture by positive distractor compared to negative and neutral task-irrelevant words has been observed in a prior study with a similar digit categorization task (Aquino and Arnell, 2007), whereas effects for negative distractor words have been reported when participants carried out a perceptual primary task (Trauer et al., 2012). Following the proposal made by Keuper et al. (2013), it may be speculated that processing requirements imposed by the primary task may determine valence-dependent effects elicited by distractor words. In this sense, the processing of positive distractor words would be more evident in tasks demanding conceptual analysis to some extend (as in the current and in Aquino and Arnell’s studies), given the greater implication of lexico-semantic processing in digit categorization tasks (see below). In contrast, activity associated with the processing of negative distractor words would be preferentially observed with primary tasks that do not require conceptual processing (e.g., the perceptual task used by Trauer et al., 2012) since the processing of negative content in words seems to rely in language-independent mechanisms according to the proposal by Keuper et al. (2013).

On another level, our results complement prior findings with CDTD tasks in several aspects. They suggest that ERP modulations triggered by task-irrelevant emotional words may emerge at different processing stages. On the one hand, in convergence with the results by Trauer et al. (2012) with a SSVEP paradigm we did not observed that emotional compared to neutral distractor words enhanced sensory processing in visual areas. This claim seems to be supported by the lack of amplitude differences in the posterior P1, which is mainly elicited in visual cortices (Di Russo et al., 2003, 2005). On the other hand, we only found modulations at early processing stages, which disagree with effects during meaning derivation – in P2 and N400 components- reported in Trauer et al.’s (2012) study. Tentatively, these discrepant results may be again related to the functionally different processes involved in the primary task in both studies (see above). In the experiment by Trauer et al. (2012), participants attended an array of squares in order to detect brief coherent movements in one direction, a task that mainly implies early perceptual processing. In contrast, we used a digit categorization task that relies on numerical skills that require more elaborated conceptual knowledge at the stage of meaning evaluation (Delazer, 2003). Thus, it could be speculated that the emotional content of word distractors interrupted ongoing task performance by capturing those processing resources that were involved to a lesser extent in the processing of target stimuli. The foreground task in our experiment may also account for the lack of effects in other components such as the EPN or the LPC. In this direction, although similar EPN modulations were found in tasks placing different processing demands, such as structural analysis or lexico-semantic processing (e.g., Kissler et al., 2009; Schacht and Sommer, 2009b), there is some evidence indicating that the EPN is more likely to be elicited when emotional words are deeply processed (e.g., Hinojosa et al., 2010b; Rellecke et al., 2011; Bayer et al., 2012). Similarly, task-effects have been found to modulate the amplitude of LPC (e.g., Fischler and Bradley, 2006; Schacht and Sommer, 2009b). Therefore, emotional modulations in these components seem to be more evident as the level of attention to the valence increases, although this idea requires further confirmation. Nonetheless, the results of a recent meta-analysis (Carretié, 2014) emphasized the nature of the primary task, as well as the characteristics of the distractors and individual differences, as a modulatory factor mediating attentional capture by emotional task-irrelevant stimuli (see also Mogg and Bradley, 1998).

The anterior N1 effects indicate that the processing of positive content in distractor words may operate at very early stages of the processing, as proposed by automatic vigilance models (Pratto and John, 1991) or the affective-primacy hypothesis (Zajonc, 1980; Delaney-Busch and Kuperberg, 2013), at least when the primary task implicates conceptual processing to some extent. However, the early latency of our effects raises the question about the mechanism underlying such a fast activation of emotional meaning from written words. Current findings suggest that some of the processes involved in word recognition become evident around 100 ms (Hauk et al., 2006). Indeed, ERP evidence has been reported suggesting a rapid access to the affective content of words as early as 80 ms using the semantic differential technique (Skrandies, 1998). Also, the finding of specific ERP effects for positive words between 100 and 150 ms with lexical decision (Bayer et al., 2012) or picture naming tasks (Hinojosa et al., 2010a) suggests that the analyses of emotional meaning has already started at 100 ms after word onset. An alternative explanation, however, might be outlined based on the proposal made by Bayer et al. (2012; see also Kissler et al., 2009, for similar arguments). These authors suggested that instead of fast semantic processing, non-linguistic mechanisms may contribute to early emotion effects in words. They argued that early emotional responses to words may originate from associative learning that does not depend on the semantic system given the results of previous studies that reported very early ERP modulations for non-linguistic stimuli associated with threat related pictures (Stolarova et al., 2006) and reward (Schacht et al., 2012). Additional support for this view, with verbal stimuli, comes from recent evidence showing that the activity elicited by emotionally and neutrally conditioned pseudowords differed in a negative component between 80 and 120 ms (Fritsch and Kuchinke, 2013). Interestingly, the OFC seems to be critically involved in rapid stimulus-reinforcement association learning for positive reinforcers (Rolls, 2000; Gottfried et al., 2003). This leaves open the possibility that associative learning mechanisms that are non-linguistic in nature underlie anterior N1 effects to positive distractor words.

The current study has several potential limitations. In this sense, the absence of jitter between the fixation cross and the stimulus onset may have increased early attentional processes as a result of the expectation generated when the cross appeared in the screen. Also, the presentation of the blank screen following rather short stimulus durations (150 ms) may have interfered, and thus interrupted, subsequent stimulus, and attentional processing. Future research can address these issues by randomly varying the time between the fixation cross and the stimulus and by directly comparing the processing of stimulus with different presentation durations. Finally, although the main focus of the current study was on early latency components (N1 and P1), the relatively high number of temporo-spatial factors that we explored may have increased the probability of finding significant effects.

In sum, several conclusions can be derived from current results. First, complementing previous findings with pictorial stimuli in CDTD tasks, our data show that salient but task-irrelevant words disrupt processes involved in a primary digit categorization task. Second, positive distractor words are able to engage automatic attentional resources at early stages of the processing, as reflected by modulations in an anterior N1 component. Third, activation of the OFC underlies exogenous attentional mechanisms devoted to the processing of task-irrelevant emotional words. Finally, the fact that attentional capture was selectively triggered by positive words emphasizes the involvement of this brain structure in the processing of positive emotion.

## AUTHOR CONTRIBUTIONS

Conception and design of the work: José A. Hinojosa and Luis Carretié. Acquisition, analysis, or interpretation of data for the work: José A. Hinojosa, Francisco Mercado, Jacobo Albert, Paloma Barjola, Irene Peláez, Cristina Villalba-García and Luis Carretié. Drafting the work or revising it critically for important intellectual content: José A. Hinojosa, Francisco Mercado, Jacobo Albert, Paloma Barjola, Irene Peláez, Cristina Villalba-García and Luis Carretié. Final approval of the version to be published: José A. Hinojosa, Francisco Mercado, Jacobo Albert, Paloma Barjola, Irene Peláez, Cristina Villalba-García and Luis Carretié. Agreement to be accountable for all aspects of the work in ensuring that questions related to the accuracy or integrity of any part of the work are appropriately investigated and resolved: José A. Hinojosa, Francisco Mercado, Jacobo Albert, Paloma Barjola, Irene Peláez, Cristina Villalba-García and Luis Carretié.

## Conflict of Interest Statement

The authors declare that the research was conducted in the absence of any commercial or financial relationships that could be construed as a potential conflict of interest.
